# Shoulder imbalance treated with scapuloplasty surgery in scoliosis patients: a clinical retrospective study

**DOI:** 10.1186/s13018-020-01676-9

**Published:** 2020-04-16

**Authors:** Rui Zhong, Zhengjun Hu, Deng Zhao, Fei Wang, Hehong Zhao, Yijian Liang

**Affiliations:** Department of Spine Surgery, the Third People’s Hospital of Chengdu, Chengdu, China

**Keywords:** Scoliosis, Shoulder imbalance, Scapuloplasty, Correction, Shoulder function

## Abstract

**Background:**

To discuss the clinical efficacy and safety of scapuloplasty treating the shoulder imbalance in scoliosis patients.

**Methods:**

A retrospective analysis was made on 21 patients who underwent scoliosis corrective surgery combined with scapuloplasty from September 2013 to March 2015. The average follow-up was 31.4 ± 5.3 months (24–42 months). The shoulder vertical difference (SVD), adjusted Constant-Murley score, range of shoulder motion function, Cavendish grade, and the overall satisfaction were compared among the pre-surgery, post-surgery, and the final follow-up periods.

**Results:**

The shoulder vertical difference (SVD) significantly decreased at the time of post-surgery and the final follow-up, comparing the score of the pre-surgery. The SVD of all patients were decreased from preoperatively 3.2 ± 1.1 cm to 0.4 ± 0.3 cm, with the Cavendish grade improved to grade 1 at the final follow-up. The adjusted Constant-Murley score and range of shoulder motion function showed no significant difference during the three time periods (*p* > 0.05). And no severe complications had occurred over 2 years follow-up.

**Conclusion:**

The scapuloplasty surgery shows to be an effective and safety procedure to improve the shoulder imbalance, cosmetic appearance, and the overall satisfaction in scoliosis patients without impairing the shoulder function, which can be widely applied in clinic.

## Background

Scoliosis is a three-dimensional structural deformity of the spine with lateral curvature more than 10° at one or more segments on the coronal plane, even with spine rotation and sagittal imbalance. In addition to causing gross truncal imbalance, it can also lead to local imbalance in the body. Such imbalances caused abnormal cosmetic appearance of the scoliosis patients, imposing huge physical and psychological burden as well as anxiety. The three main cosmetic appearance abnormalities which the patients most concerned about and prompted their treatment are shoulder imbalance, razor back and waist angulation [1–3]. Therefore, the shoulder balance is not only an important aspect of evaluating the cosmetic appearance of scoliosis patients, but also a primary indicator for assessing clinical efficacy after corrective surgery [4]. However, in clinical practice, we have found that even if the patients may successfully achieve the gross body balance according to radiological parameters after a well-designed deformity surgery, some patients still remained unbalanced shoulder postoperatively, which can significantly worsen the outcome of the surgery and the overall satisfaction of patients [5, 6]. Thus, the scapuloplasty surgery might contribute to address the residual shoulder imbalance in patients who underwent deformity surgery, so as to achieve better cosmetic appearance and improve patient satisfaction. Accordingly, the aim of this study was to retrospectively analyze patients undergoing scoliosis correction surgery combined with scapuloplasty in our hospital, and comprehensively evaluate the clinical efficacy of scapuloplasty from the aspects of cosmetic appearance, shoulder joint function, and patient satisfaction for more than 2 years follow-up.

## Methods

### General information

A retrospective analysis of patients who underwent scoliosis correction and scapuloplasty surgery between September 2013 and March 2015 in our hospital was carried out. The inclusion criteria were as follows: (1) suffered from scoliosis and intended to undergo correction surgery owing to spinal deformity; (2) with preoperative elevated shoulder which the difference less than 5 cm(Cavendish score from 2 to 3 points); (3) a minimum of 2 years follow-up time with complete clinical data; (4) without malformation of scapula and previous shoulder surgery; (5) underwent scoliosis correction combined with scapuloplasty surgery in one stage. The exclusion criteria were as follows: (1) a history of a spinal or shoulder surgery; (2) with significant elevated shoulder which the difference was more than 5 cm (Cavendish score 4 points); (3) a severe concomitant disease, such as congenital malformation of scapula or infection. All patients were operated on by the same spine surgeon. A total of 21 patients (14 females and 7 males) were included, with an average age of 19.6 ± 3.6 years old (range 14–28) and an average follow-up time 31.4 ± 5.3 months (range 24–42).

### Evaluation indicators

**Fig. 1:**
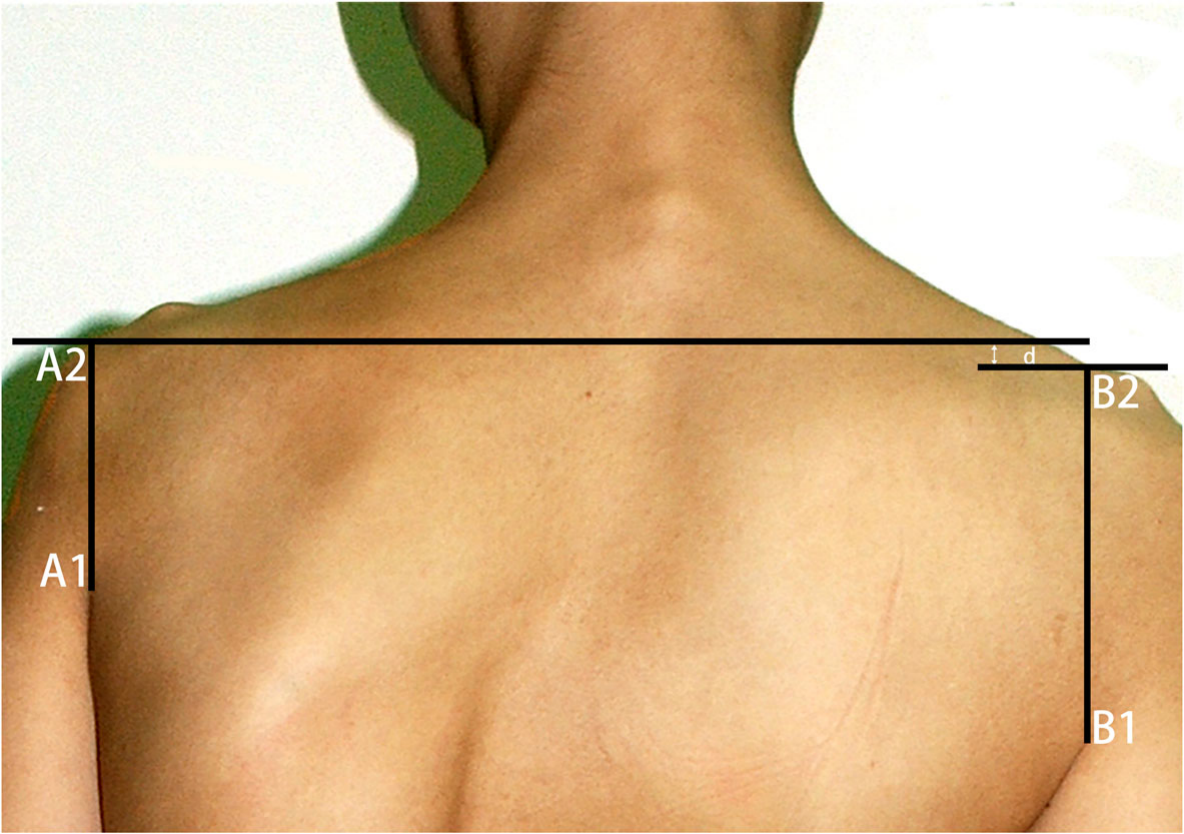
SVD measurement diagram. SVD was the height difference between the horizontal lines where vertical lines A1–A2, B1–B2 intersected with the shoulders d

In this retrospective study, the patient data of the shoulder vertical difference (SVD), adjusted Constant-Murley score, range of shoulder motion function, Cavendish score, and overall satisfaction were collected and evaluated preoperatively, postoperatively, and at the latest follow-up. (1) Shoulder vertical difference (SVD) is measured according to the method which introduced by Qiu, Akel et al. [4, 7] as below: two vertical lines are drawn from the bilateral vertex of axillary folds (A1, B1) to the upper edge of the shoulder (the intersection points are marked as A2, B2). The perpendicular height difference between the plumb lines to the line A1–A2 and B1–B2 is known as shoulder vertical difference (SVD) (Fig. 1). (2) Adjusted Constant-Murley score is currently widely used for assessing the shoulder function. It consists of both subjective and objective items with a total score of 75 points, which a maximum of 15 points are given to pain, 20 for daily activities, and 40 for painless shoulder motion function. The higher the adjusted Constant-Murley score, the better the overall shoulder function. (3) The shoulder motion function is evaluated by the 4 major activities of shoulders (flexion, extension, abduction, and adduction, respectively), which the maximum range of painless shoulder motion is compared. (4) The Cavendish score [8] is used to grade the cosmetic appearance with a total of 4 scores, 1 score: very mild, shoulder joints are level, deformity is invisible when patient is dressed; 2 score: mild, elevated shoulder is visible when dressed; 3 score: moderate, shoulder elevation 2-5 cm which is easily visible; 4 score: severe, shoulder elevation exceeds 5cm. Furthermore, patients were asked to rate the overall satisfaction in terms of “bad,” “fair,” or “good.”

### Surgical technique

**Fig. 2:**
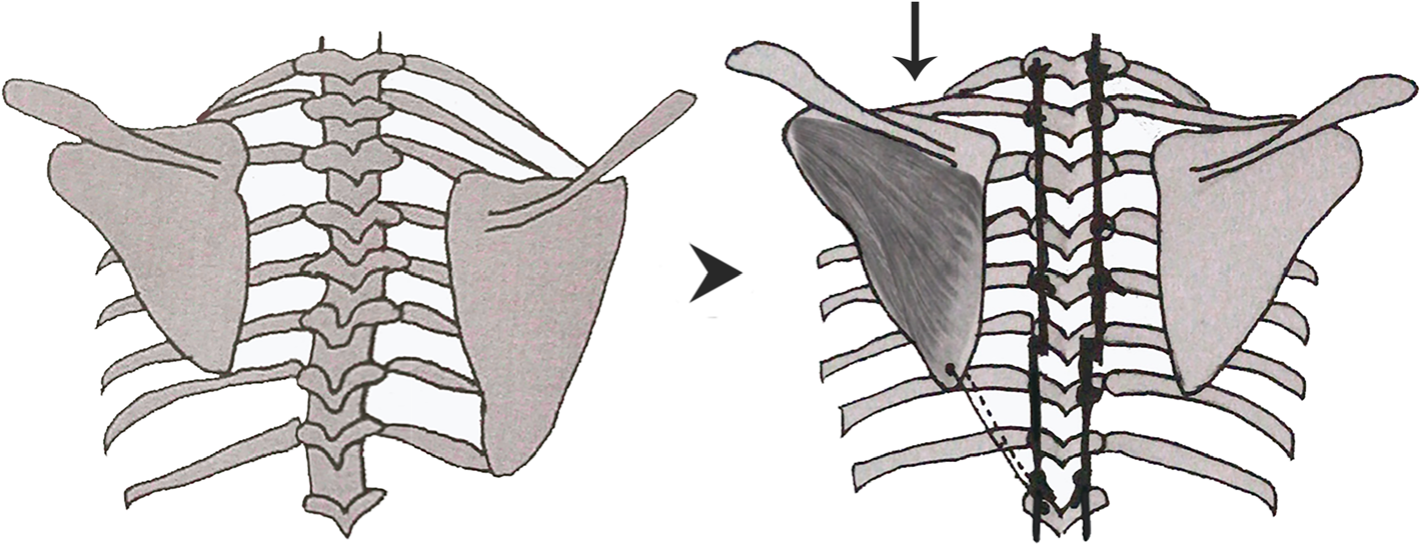
The diagram of scapuloplasty

**Fig. 3:**
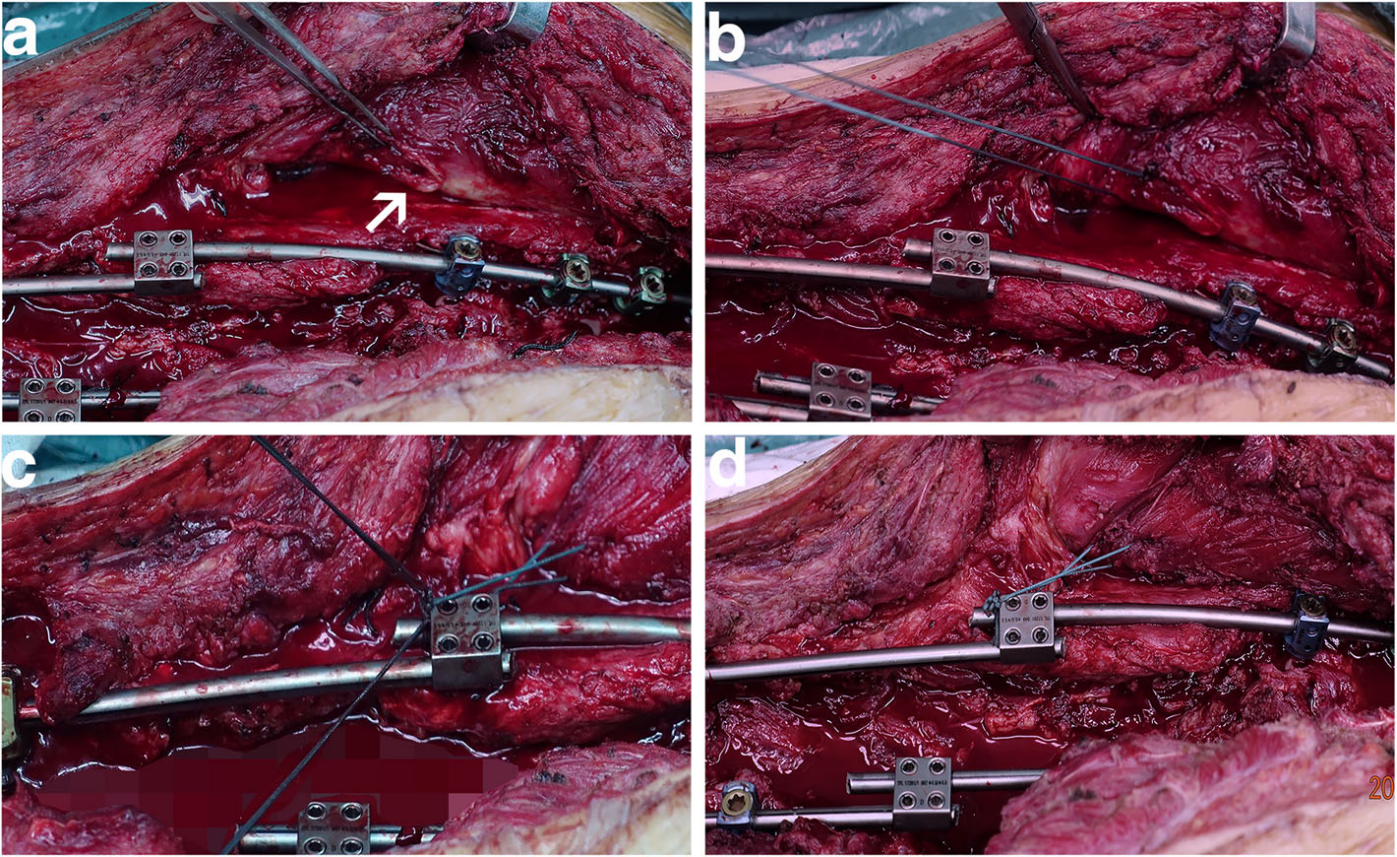
Illustration of key step of the scapuloplasty. White arrows indicated the muscle sheath covered by the scapula. **a** Exposed the muscle sheath. **b**–**d** The muscle sheath anchored by the tendon suture on homolateral titanium rod

The surgery was briefly illustrated by the diagram (Fig. 2). Scapuloplasty started while the spinal correction procedure was completed, using the same midline incision. Firstly, make sure which scapula was elevated through the transparent surgical drape. The attachments of the aponeurosis of the muscles which covering the inferior medial angle of the elevated scapula were detached. And the surrounding adhesion tissue between the scapula and the chest wall was also removed. The scapula could then be relocated to the same level of the contralateral scapular spine by gently pushing it caudal on the thorax. exposing the sheath of muscles covered by the inferior medial angle of the scapula, which is mainly composed of the subscapular muscle and infraspinatus muscle (Fig. 3a). Subsequently, the muscle sheath were sutured and fixed on the same side of the titanium rod by using 2–3 stitches of tendon sutures(Ethibond Excel MB66)(Fig. 3b–d). Finally, the wound was rinsed, sutured and bandaged.

### Statistical analysis

Statistical analysis was performed with the use of SPSS 18.0 (SPSS Inc, Chicago, IL, USA). All indicators were measured by a senior spinal surgeon who was not involved in the surgery. Each indicator was measured three times and averaged. All measurement data were expressed as mean ± standard deviation (SD). We used the one-way ANOVA analysis to compare the data of SVD, range of shoulder motion, adjusted Constant-Murley score and Cavendish score preoperatively, postoperatively, and at the latest follow-up. If the difference was statistically significant, further comparisons were performed at each time point based on the Bonferroni method (homogeneity of variance) or Tamhane method (heterogeneity of variance). Statistical significance was indicated at *p* < 0.05.

## Results

**Fig. 4:**
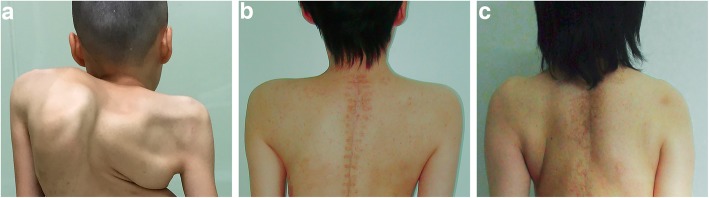
The cosmetic appearance of patient before the scapuloplasty (**a**), after surgery (**b**), at the final follow-up period (**c**)

No postoperative nerve palsies and severe complications were recorded. Cosmetic appearance had improved in all patients. After scapuloplasty, the elevated shoulder of the patients significantly decreased, substantially achieving the shoulder balance. And the level shoulder showed no significant change at the latest follow-up (Fig. 4).

### Shoulder vertical difference

**Fig. 5:**
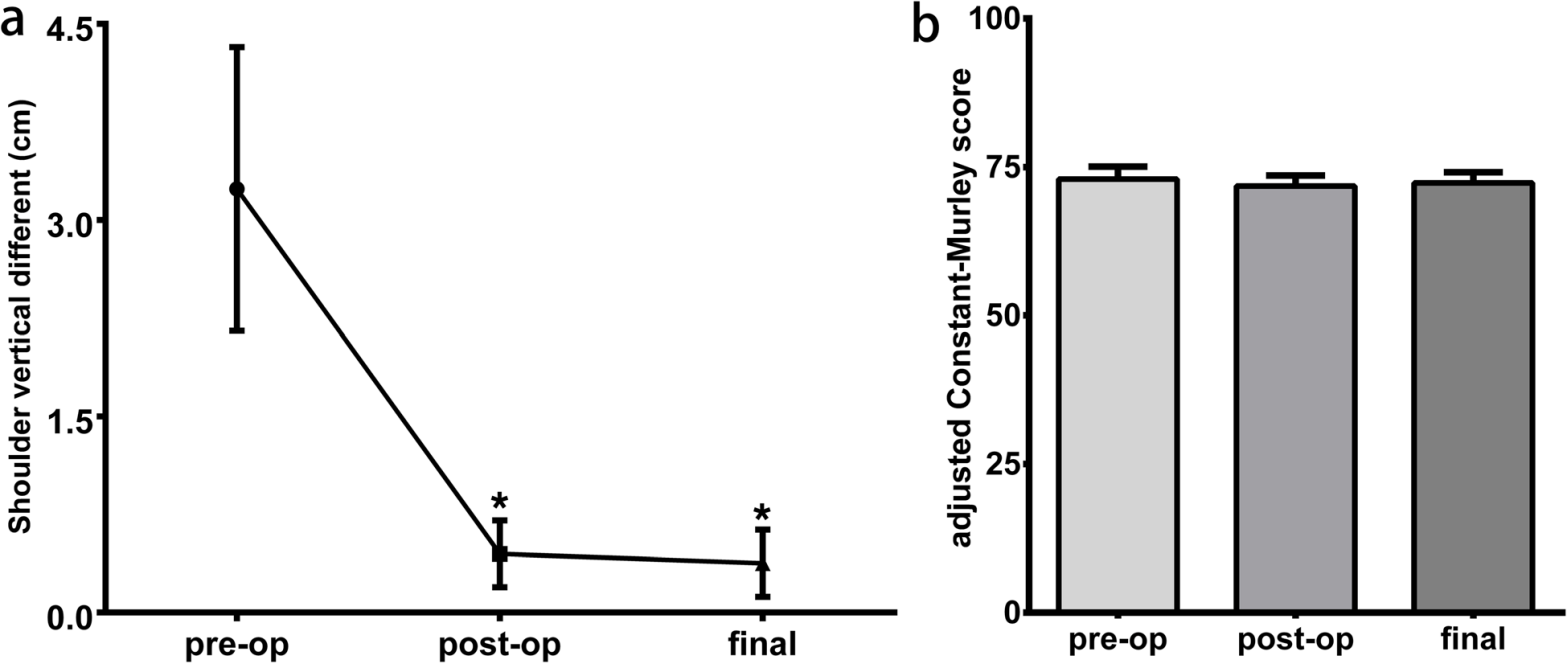
The comparisons of SVD (**a**) and adjusted Constant score (**b**) among pre-operation, post-operation, and final follow-up. * indicated *p* < 0.05 comparing with preoperative value

The mean SVD of the patients before surgery was 3.2 ± 1.1 cm (range 1.5–5), which significantly decreased to 0.4 ± 0.2 cm postoperatively, and 0.4 ± 0.3 cm at the latest follow-up. The results of one-way ANOVA showed that there was a significant difference in the SVD among preoperative, postoperative, and the latest follow-up periods (*p* < 0.05). The following Tamhane tests were applied for the comparison of every two time points, which revealed that the SVD in the postoperative and the latest follow-up periods significantly decreased compared with that of the preoperative period (*p* < 0.05). There was no significant in the SVD between the postoperative and the latest follow-up (*p* = 0.75), as shown in Fig. 5a.

### The adjusted Constant-Murley score

The adjusted Constant-Murley score were 73 ± 2.1 points, 71.8 ± 1.8, and 72.3 ± 1.8 preoperatively, postoperatively, and at the latest follow-up, respectively. The results of one-way ANOVA showed that there was no significant difference in the adjusted Constant-Murley score among 3 time points (*p* = 0.14, Fig. 5b).

### Shoulder motion function

**Fig. 6:**
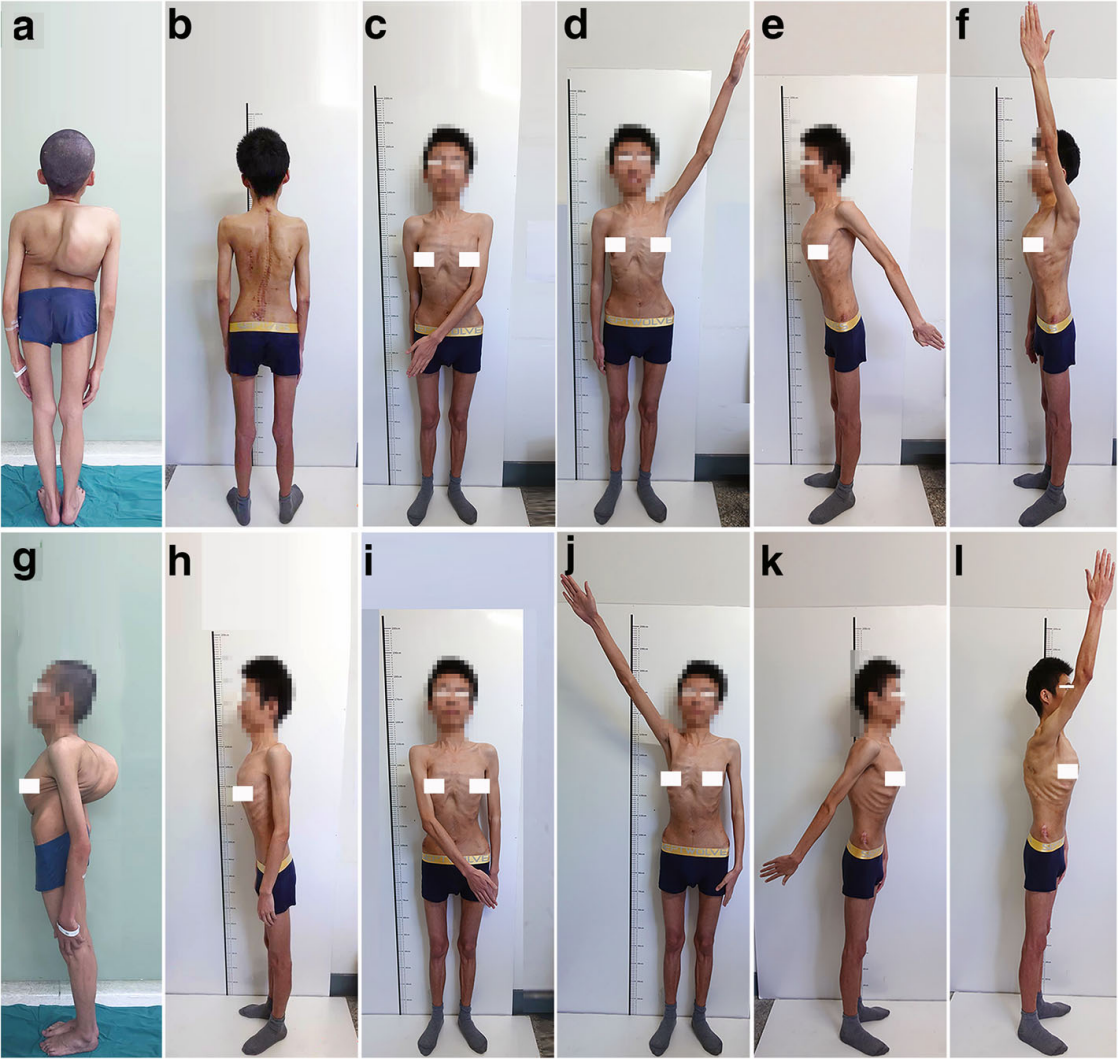
The photograph the patient. **a**, **g** The front and lateral position of the patient before the surgery. **b**, **h** The front and lateral position of the patient at final follow-up. **c**–**f** The adduction, abduction, extension and flexion of the left shoulder joint at the final follow-up. **i**–**l** The adduction, abduction, extension, and flexion of the right shoulder joint at the final follow-up

The comparison of shoulder motion function among 3 time points was shown in Tables 1 and 2. These results showed that there was no significant difference in the active range of shoulder motion over time, including flexion, extension, adduction, and abduction (*p* > 0.05). The panoramic photos of the patient at the latest follow-up showed that the elevated shoulder significantly decreased and maintained the same overall shoulder function compared with that of the preoperative period (Fig. 6).

**Table 1:** Patient characteristics

Case	Gender	Age (years)	Cavendish grade	Elevated shoulder	Follow-up (m)
1	F	15	3	Right	42
2	M	22	3	Left	40
3	F	21	2	Right	40
4	F	18	3	Left	39
5	F	16	3	Right	34
6	M	16	3	Right	34
7	F	18	3	Right	33
8	M	26	3	Left	33
9	F	20	2	Left	32
10	M	28	2	Right	31
11	F	20	3	Left	31
12	M	25	3	Left	30
13	F	22	3	Right	29
14	F	21	2	Left	29
15	F	17	3	Right	28
16	F	21	3	Right	27
17	M	14	3	Left	27
18	F	18	3	Right	26
19	F	16	3	Right	26
20	M	19	2	Right	24
21	F	19	3	Left	24

**Table 2:** The comparison of shoulder joint motion preoperatively, postoperatively, and at the latest follow-up

Shoulder joint motion	Pre-op (°)	Post-op (°)	Final (°)	*p* value
Flexion	153.2 ± 17.4	152.3 ± 16.3	153.7 ± 15.7	0.963
Extension	37.5 ± 1.60	38.0 ± 2.1	37.5 ± 1.8	0.655
Adduction	33.4 ± 3.8	32.5 ± 3.6	33.8 ± 4.4	0.533
Abduction	169.6 ± 7.7	168.2 ± 7.0	169.1 ± 8.2	0.851

### The Cavendish scores and patient satisfaction

**Fig. 7:**
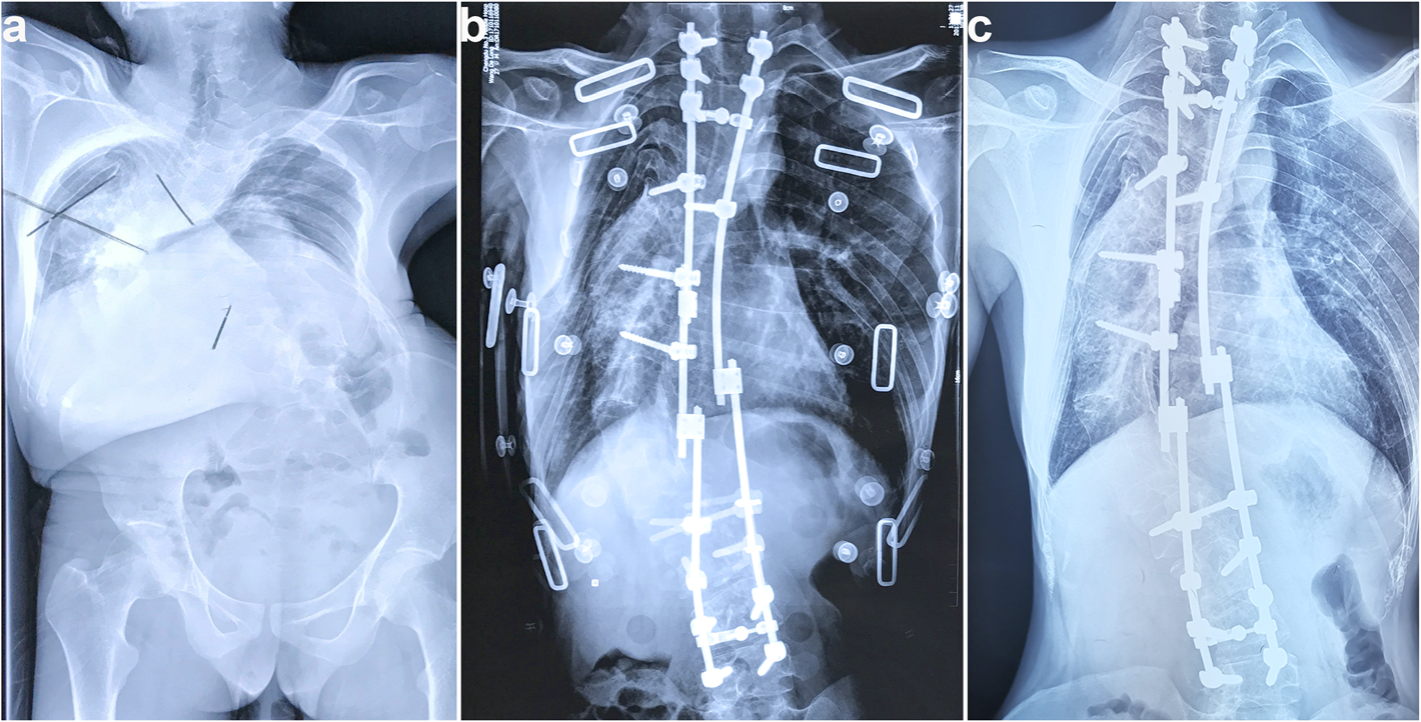
The X-ray images of patient before the scapuloplasty (**a**), after surgery (**b**), at the final follow-up period (**c**).

The Cavendish scores of preoperative patients was 3 point, except for 2 point in 4 patients. Whereas the scores of the postoperative and final follow-up were reduced to 1 point for all patients, that is, the deformity would not be visible when patient was dressed. The overall satisfaction concerning the results of the scapuloplasty procedure was rated “good” at the last follow-up in all 21 patients. Also, the X-ray showed the radiological shoulder balance was achieved post-surgery and at the final follow-up (Fig. 7).

## Discussion

In addition to the gross truncal imbalance caused by scoliosis, the significant local body imbalance is also the primary reason for the patient to seek treatment. The treatment of scoliosis has improved considerably over the past several years due to advancements in spinal instrumentation and surgical procedures. And the main concern about treatment is still how to achieve the gross truncal balance currently, however, the local body imbalances such as shoulder imbalance also need to be drawn attention for spine surgeon. Moreover, even if the surgeon achieves the excellent gross body balance in terms of radiological assessment, shoulder imbalance can still remain. Asymmetrical shoulder is more likely to be found in cosmetic appearance than the razor back when patient is dressed, which can significantly worsen the overall patient satisfaction. We believed the spinal deformity correction surgery should not only focus on simple spinal fusion, but should also seek to reconstruct the aesthetic appearance of scoliosis patient. In our experience, there are three aesthetic appearance balances should be achieved after correction surgery: shoulder balance, back balance and pelvis balance. The concept of three aesthetic appearance balances proposed by our department means that there would be no visible elevated shoulder, razor back, and tilt pelvis after deformity surgery, achieving the best aesthetic appearance of scoliosis patients. Therefore, it is of great significance for patients with imbalanced shoulders after spinal deformity surgery to achieve the shoulder balance. However, there is limited research focusing on the residual shoulder imbalance worldwide right now. The correction of elevated shoulders is still mainly concentrated in patients with congenital Sprengel’s deformity. The cosmetic malformation and impairment of shoulder abduction are the 2 main problems in Sprengel’s deformity patients which caused by hypoplastic scapula. Currently, conservative treatment has been proven to be ineffective for this deformity [9]. Therefore, several surgical procedures have been developed to address these problems, including the Woodward procedure and Green surgical procedures, which are the two classic surgical technique for the treatment of Sprengel’s deformity [10, 11]. The disadvantages of Green’s surgical procedure include the large trauma, the brachial plexus injury caused by excessive stretching during the operation and the limited range of the shoulder motion which might result from the detachment of the muscles. Although the clinical efficacy of Woodward is better than that of Green’s procedure, there are still some disadvantages such as requirement of extra external fixation postoperatively and correction loss over time [12–14]. Therefore, it is necessary to seek a new procedure with less trauma, simple operation, reliable correction effect, and without impairment of the shoulder motion function, in order to solve the residual shoulder imbalance after correction surgery.

In the scapuloplasty procedure proposed by our department, the elevated scapula is mobilized distally to approximately the same level of the contralateral scapular spine and fixed on the same side of the titanium rod with normal tendon suture. The advantages of this surgery include short operation time, less bleeding, no need of special equipment, and limit impairment of the muscles around the scapula. We sutured the inferior medial angle of muscle sheath of the scapula, which is mainly composed of the subscapular muscle and infraspinatus muscle, to the titanium rod so as to firmly maintain the new position of the scapula. Subscapular muscle underlying the scapula is innervated by the subscapular nerves, mainly contributes to the adduction, internal rotation and posterior extension of the shoulder joint. Infraspinatus muscle which occupies the chief part of the infraspinous fossa is innervated by the suprascapular nerve mainly acts on the abduction and external rotation of the shoulder joint. However, the adduction, internal rotation, extension, and external rotation of the shoulder joint are also affected by the teres major and teres minor. Therefore, fixing the muscle sheath of partial subscapular muscle and infraspinatus muscle distally to the titanium rod has little effect on the motion function of the shoulder joint. Falla and Kibler et al. [14, 15] reported that the trapezius, rhomboids, and levator scapulae play a major role in the scapular motility and maintain its stability. Our procedure also did not impair the trapezius, rhomboids, or levator scapulae. Therefore, there is no negative effect on the flexion, extension, adduction, and abduction of the scapula. Our results also showed there is no significant deterioration in the range of shoulder motion in terms of flexion, extension, abduction, and adduction over 2 years follow-up.

There is still controversy about the criteria of shoulder balance. The shoulder height difference greater than 1 cm is mostly defined as shoulder imbalance, while the shoulder height difference > 2 cm is thought to be obviously unbalanced [5, 16, 17]. Akel et al. found that the shoulder height difference ≤ 1.5 cm could exist in normal adolescents [16]. In addition, some researchers proposed that the asymmetrical shoulder heights could be noticed visually when the height difference is greater than 0.5 cm. Therefore, they believed that the shoulder height difference should be within 0.5 cm for the ideal shoulder balance. In the present study, we demonstrated that the scapuloplasty used in our department can significantly decrease the SVD and improve the cosmetic appearance of all patients. The SVD was controlled within 1cm postoperatively and at the last follow-up. Moreover, 66.7% (14/21) of the patients had an SVD of less than 0.5 cm at the last follow-up, achieving the best aesthetic cosmetic appearance.

The concept of proximal thoracic curve was first proposed by Ponseti et al. in 1950. Yet, the indications of proximal thoracic curve fusion and the selection of the upper instrumented vertebra are still controversial while correction of the main curvature. Currently, it is believed that the shoulder imbalance is closely related to proximal thoracic curve, and the selection of the upper fusion vertebra in this region has a direct impact on the shoulder balance and cosmetic appearance of the patients underwent deformity corrective surgery. Suk et al. [6] reported that idiopathic thoracic scoliosis with a proximal thoracic curve of more than 25° and a level or elevated left shoulder should be treated with fusion of upper thoracic curve; otherwise, it may lead to postoperative shoulder imbalances. However, some studies have shown that even though the surgeon can achieve the successful correction of the proximal thoracic curvature, shoulder imbalance can still remain regardless of the amount of the Cobb angle correction, which can significantly worsen the outcome of the surgery [18]. Qiu et al. [19] also pointed out that there was a high risk of postoperative shoulder imbalance when the patient with elevated left shoulder, low flexibility of upper thoracic curve, and large Cobb angle of main curvature before surgery. According to our study, scapuloplasty surgery can finally achieve shoulder balance in patients who still have shoulder imbalance after the fusion of the upper thoracic curve, which would improve the cosmetic appearance and patient satisfaction.

The most serious complication of scapuloplasty is the brachial plexus injury resulting from inferior displacement of the scapula. Some researchers found that children who are 8 years of age and older is more likely to suffer brachial plexus injury due to the very limited anatomy elasticity. However, all 21 patients (> 8 years old) in our study had no brachial plexus injury, which mainly due to the fact that we gradually mobilized the scapula distally with a gentle force and used intraoperative somatosensory evoked potential monitoring during the traction process [10].

The present study also has limitations. The sample size was relatively small and from a single center. Also, retrospective studies suffer from a number of inherent limitations like selection bias. Additionally, the scapuloplasty surgery might have a “ceiling effect,” which means the elevated scapula could only be mobilized maximally 5 cm to achieve shoulder balance in our experience. Therefore, the patients with shoulder difference more than 5 cm (Cavendish score 4 points) are not indicated for the scapuloplasty surgery. We would figure out another method to address such patient in the future.

## Conclusion

The scapuloplasty combined with corrective surgery shows to be an effective and safety procedure to address the residual shoulder imbalance and improve the cosmetic appearance and the overall satisfaction without impairing the shoulder motion function. The clinical efficacy of this procedure remains stable without severe complications over 2 years follow-up, which indicates that scapuloplasty might be widely applied in clinic.

## Data Availability

The data and materials contributing to this article may be made available upon request by sending an e-mail to the first author.

## Abbreviations

SVD: The shoulder vertical difference

## References

[1] Raso VJ, Russell GG, Hill DL (1991). Thoracic lordosis in idiopathic scoliosis[J]. J Pediatr Orthop.

[2] Kuklo TR, Lenke LG, Graham EJ (2002). Correlation of radiographic, clinical, and patient assessment of shoulder balance following fusion versus nonfusion of the proximal thoracic curve in adolescent idiopathic scoliosis[J]. Spine (Phila Pa 1976).

[3] Sharma S, Andersen T, Wu C (2016). How well do Radiologic Assessments of Truncal and Shoulder Balance Correlate with Cosmetic Assessment Indices in Lenke 1C Adolescent Idiopathic Scoliosis? [J]. Clin Spine Surg.

[4] Akel I, Pekmezci M, Hayran M (2008). Evaluation of shoulder balance in the normal adolescent population and its correlation with radiological parameters[J]. Eur Spine J.

[5] Li M, Gu S, Ni J (2009). Shoulder balance after surgery in patients with Lenke Type 2 scoliosis corrected with the segmental pedicle screw technique[J]. J Neurosurg Spine.

[6] Suk SI, Kim WJ, Lee CS (2000). Indications of proximal thoracic curve fusion in thoracic adolescent idiopathic scoliosis: recognition and treatment of double thoracic curve pattern in adolescent idiopathic scoliosis treated with segmental instrumentation[J]. Spine (Phila Pa 1976).

[7] Qiu XS, Ma WW, Li WG (2009). Discrepancy between radiographic shoulder balance and cosmetic shoulder balance in adolescent idiopathic scoliosis patients with double thoracic curve[J]. Eur Spine J.

[8] Cavendish ME (1972). Congenital elevation of the scapula[J]. J Bone Joint Surg Br.

[9] Khairouni A, Bensahel H, Csukonyi Z (2002). Congenital high scapula[J]. J Pediatr Orthop B.

[10] Farsetti P, Weinstein SL, Caterini R (2003). Sprengel’s deformity: long-term follow-up study of 22 cases[J]. J Pediatr Orthop B.

[11] Aydinli U, Ozturk C, Akesen B (2005). Surgical treatment of sprengel's deformity: a modified Green procedure[J]. Acta Orthop Belg.

[12] Schrock RD (2008). Congenital elevation of the scapula: Robert D. Schrock MD (1884-1960). The 9th president of the AAOS 1940[J]. Clin Orthop Relat Res.

[13] Mears DC (2001). Partial resection of the scapula and a release of the long head of triceps for the management of Sprengel's deformity[J]. J Pediatr Orthop.

[14] Kibler WB (1998). The role of the scapula in athletic shoulder function[J]. Am J Sports Med.

[15] Falla D, Bilenkij G, Jull G (2004). Patients with chronic neck pain demonstrate altered patterns of muscle activation during performance of a functional upper limb task[J]. Spine (Phila Pa 1976).

[16] Kuklo TR, Lenke LG, Won DS (2001). Spontaneous proximal thoracic curve correction after isolated fusion of the main thoracic curve in adolescent idiopathic scoliosis[J]. Spine (Phila Pa 1976).

[17] Smyrnis PN, Sekouris N, Papadopoulos G (2009). Surgical assessment of the proximal thoracic curve in adolescent idiopathic scoliosis[J]. Eur Spine J.

[18] Sucato DJ, McClung A (2008). Inclusion of the proximal thoracic curve does not provide better shoulder balance in all Lenke 2 curves (Paper #9) [C].

[19] Qiu Y, Qiu XS, Ma WW (2009). Double thoracic adolescent idiopathic scoliosis:clinical evaluation of shoulder balance [J]. Chin J orthop.

